# Association between new Life’s Essential 8 and the risk of all-cause and cardiovascular mortality in patients with hypertension: a cohort study

**DOI:** 10.1186/s12889-024-19189-z

**Published:** 2024-06-28

**Authors:** Lu He, Miao Zhang, Yang Zhao, Wei Li, Yushun Zhang

**Affiliations:** 1 Department of Structural Heart Disease, The First Affiliated Hospital of Xi’an Jiaotong University, No.277, Yanta West Road, Xi’an, Shaanxi 710061 PR China; 2 Department of Cardiovascular Medicine, Weinan Central Hospital, Weinan, 714000 China; 3 Department of Cardiovascular Medicine, 521 Hospital of Norinco Group, Xi’an, 710061 China

**Keywords:** Cardiovascular health, Life’s Essential 8, Hypertension, Mortality, Cohort study

## Abstract

**Background:**

The American Heart Association recently introduced a new model for cardiovascular health (CVH) known as Life’s Essential 8 (LE8). The impact of LE8 on hypertensive individuals is currently unclear. In our study, we investigated the correlation between comprehensive and individual CVH indicators as defined by LE8, and the mortality rates in hypertension patients.

**Methods:**

We analyzed a total of 8,448 hypertensive individuals aged ≥ 20 years who participated in the National Health and Nutrition Examination Survey from 2007 to 2016. These participants were nonpregnant and noninstitutionalized. We identified their mortality by linking their data to the National Death Index until December 31, 2019. The overall cardiovascular health (CVH) was assessed using the LE8 score, which ranged from 0 to 100. Additionally, we evaluated the scores for each component of diet, physical activity, tobacco/nicotine exposure, sleep duration, body mass index, non-high-density lipoprotein cholesterol, blood glucose, and blood pressure. The CVH were categorized into low (0–49), moderate (50–79), and high (80–100) CVH.

**Results:**

Over an average follow-up period of 7.41 years, 1,482 (17.54%) of the participants died, among which 472 deaths were attributed to CVD. When compared to adults with lower total CVH scores, those with elevated total CVH scores displayed a 37% reduced risk of mortality from all causes (adjusted hazard ratio [aHR] = 0.63, 95% confidence interval [CI] = 0.45–0.88). In relation to CVD-specific mortality, the corresponding aHRs for moderate and high total CVH scores were 0.76 (0.60–0.97) and 0.54 (0.31–0.94), respectively. Furthermore, after adjusting for potential confounders, it was observed that higher scores on the LE8 index were associated with a reduced risk of both all-cause mortality (aHR for every 10-score increase, 0.91; 95% CI = 0.86–0.96) and CVD-specific mortality (aHR for every 10-score increase, 0.82; 95% CI = 0.75–0.90). Notably, a linear dose–response relationship was observed in this association. Similar patterns were identified in the relationship between health behavior and both all-cause and CVD-specific mortality.

**Conclusions:**

Achieving a higher CVH score, as per the new LE8 guidelines, has been found to be associated with a reduced risk of mortality from all causes and specifically from CVD in patients with hypertension. Therefore, public health and healthcare initiatives that focus on promoting higher CVH scores could potentially yield significant benefits in terms of reducing mortality rates among individuals with hypertension.

**Supplementary Information:**

The online version contains supplementary material available at 10.1186/s12889-024-19189-z.

## Introduction

Hypertension, affecting approximately 1.13 billion individuals worldwide, is a significant global health concern [1]. Hypertension remains the leading cause of cardiovascular events and mortality across the world [2]. As a primary contributor to cardiovascular diseases (CVDs), it significantly adds to global morbidity and mortality [3]. There has been substantial progress in developing pharmacological interventions for hypertension; however, managing hypertension effectively requires a multi-faceted approach. Non-pharmacological interventions have been advocated as vital components of hypertension management. These strategies are often multidimensional, involving dietary modifications, physical activity enhancement, weight management, moderation in alcohol consumption, and cessation of smoking [4].

The American Heart Association's Life's Essential 7, a suite of modifiable lifestyle factors comprising smoking status, physical activity, diet, body mass index, blood pressure, cholesterol, and blood glucose levels, has been widely utilized in clinical practice [5, 6]. Recently, based on self-reported average hours of sleep per night, sleep health has been integrated into this initiative called Life's Essential 8 (LE8) [7]. This emphasizes the crucial role of sleep health in general well-being and cardiovascular health, recognizing the established association between poor sleep and heightened cardiovascular risks [8].

Extensive evidence has confirmed that the ideal cardiovascular health (CVH), as defined by LE8, is associated with increased survival free from CVD, overall longevity, and improved quality of life [7, 9–11]. However, there is still a limited number of studies that explore the association between LE8 and all-cause and cardiovascular mortality in patients with hypertension. This prospective cohort study, using the National Health and Nutrition Examination Surveys (NHANES) data, aims to address this gap, exploring the association between LE8 and the risk of all-cause and cardiovascular mortality in hypertensive patients.

## Methods

### Study population

The NHANES is an ongoing, nationally representative study in the United States that tracks participants biennially since 1999, accumulating data on the health and nutritional conditions of non-institutionalized US citizens. The protocol of the NHANES study received approval from the Research Ethics Review Committee of the National Centers for Health Statistics (NCHS), and each participant provided their written informed consent. Interviews take place in the homes of the participants, which are then followed by examinations and lab tests performed in mobile examination centers. The study collected information on demographic characteristics, dietary habits, physical health assessments, and questionnaire responses. Skilled interviewers conducted an in-home interview and obtained automated data.

**Fig. 1:**
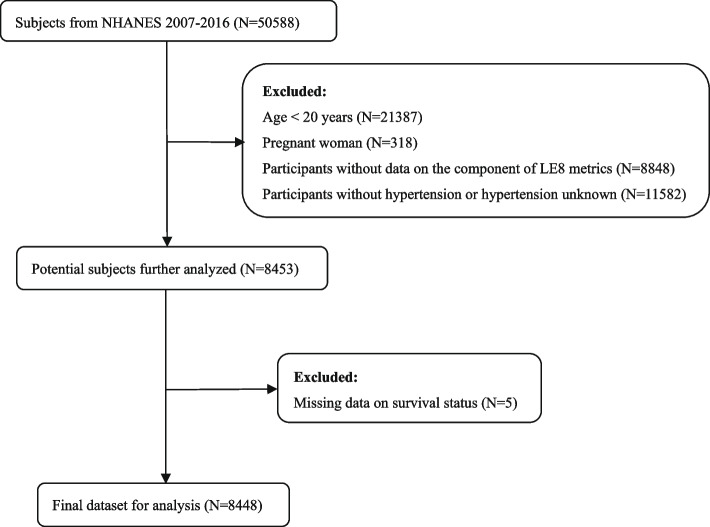
Flow chart of the sample collection in this study

The data for this study was sourced from five continuous NHANES cycles from 2007 to 2016. A total of 50,588 participants were initial included. Exclusions were made for individuals under the age of 20, pregnant individuals, and those lacking data on the LE8 metrics components, as well as participants without hypertension or unknown hypertension status. After removing 5 missing deaths, the study included a total of 8,448 patients (Fig. 1**)**.

### Assessments of CVH

The LE8 scoring algorithm comprises four health behaviors (diet, physical activity, nicotine exposure, and sleep duration) and four health factors (body mass index [BMI], non-high-density lipoprotein cholesterol, blood glucose, and blood pressure). Detailed algorithms for calculating the LE8 scores for each metric using NHANES data have been previously published and can be found in Table S1. Briefly, each of the eight CVH metrics was assigned a score ranging from 0 to 100 points. The overall LE8 score was calculated as the unweighted average of these eight metrics. Each individual’s score for each of the 8 CVH metrics was determined on a scale of 0 to 100 points using the American Heart Association (AHA) algorithm. The overall CVH score for each individual was calculated by adding up the scores for each of the 8 metrics and then dividing the total by 8, resulting in an LE8 score ranging from 0 to 100. Participants with an LE8 score of 80–100 were classified as having high CVH, scores of 50–79 indicated moderate CVH, and scores of 0–49 indicated low CVH [7].

### Definition of hypertension

In accordance with the blood pressure measurement protocol established by the AHA, a trained examiner recorded the blood pressure. The average systolic and diastolic blood pressure values were obtained by taking three consecutive measurements and reported accordingly. If the patient has multiple blood pressure readings, the average is utilized to diagnose hypertension. Consistent with previous research analyzing the NHANES database, hypertension was defined as meeting any of the following criteria: (1) average systolic blood pressure (SBP) ≥ 140 mmHg, (2) average diastolic blood pressure (DBP) ≥ 90 mmHg, (3) self-reported hypertension, or (4) individuals taking prescribed antihypertensive medications. The threshold of 140/90 mmHg aligns with the guideline set by the International Society of Hypertension.

### Definitions of variables of interest

In this study, we selected a priori covariates based on clinical relevance and previously published research. Demographic variables measured using the self-reported questionnaire included age, sex, and race and ethnicity (Mexican American, non-Hispanic Black, non-Hispanic White, and Other). Levels of educational attainment were classified into three levels: less than high school, high school or equivalent, and high school above. The poverty income ratio (PIR) is an indicator that measures the ratio of household income to the poverty threshold and are classified as PIR ≤ 1.3, 1.3 < PIR ≤ 3.5, and PIR > 3.5. Marital status was categorized as unmarried and married. Individuals who have smoked less than 100 cigarettes throughout their life are categorized as never smokers. People who have smoked more than 100 cigarettes throughout their life are deemed as current smokers, while those who have smoked more than 100 cigarettes but have since stopped are identified as former smokers. Self-reported CVD diseases included angina, congestive heart failure, coronary heart disease, myocardial infarction, and stroke. History of malignancy was obtained by questionnaire. Examination and laboratory measurements consisted of BMI, waist circumference, SBP, and DBP. Diabetes was categorized based on criteria that included a patient's self-reported diagnosis, a fasting plasma glucose level equal to or exceeding 7.0 mmol/L, an HbA1c concentration of 6.5% or above, or the use of medication for blood glucose control. The use of medications such as antihypertensive drugs, antidiabetic medications, and statins was also documented.

### Ascertainment of mortality

The death status and cause of death were established by linking to the NHANES with the National Death Index's public access files up until December 31, 2019. The International Classification of Disease (ICD) was used to specify the cause of death. Mortality due to CVD was characterized as deaths caused by heart diseases (ICD-10 codes I00-I09, I11, I13, I20-I51) and cerebrovascular diseases (ICD-10 codes I60-I69).

### Statistical analysis

The NHANES uses design weighting to produce accurate national estimates. Baseline characteristics of the study population were stratified by CVH categories, with continuous variables presented as survey-weighted mean and categorical variables presented as survey-weighted percentage (%), with corresponding confidence intervals (CIs). We used the Variance Inflation Factor (VIF) to evaluate multicollinearity among all variables. Any covariates that had a VIF exceeding 5 were eliminated from our consideration. Variables with a missing value of more than 10% were only used for statistical analysis and were not included in logistic regression analysis. For each category of CVH level, we calculated age-standardized mortality estimates along with their 95% CIs. Kaplan–Meier plots were generated to display mortality risk by CVH categories. We adopted multivariate Cox proportional hazards regression to generate hazard ratios (HRs) and 95% CIs of all-cause and CVD mortality with the low CVH category as a reference. A potential variable was incorporated if it was either associated with all-cause mortality or resulted in a change of more than 10% in any effect measure [12]. Three multivariate COX regression model was developed. Model 1 was a crude model unadjusted for potential confounders. Model 2 was adjusted for sex, age, race/ethnicity, education level, marital status, PIR, BMI, waist circumference. Model 3 was further adjusted for history of malignancy, history of CVD, history of diabetes, smoking status, DBP, and SBP. The possible modifications of the association between LE8 and all-cause mortality were performed in several subgroups. We explored the relationship between LE8 and all-cause mortality in different subgroups including age (< 60 years, ≥ 60 years), sex, race (Mexican American, non-Hispanic Black, non-Hispanic White, and Other), BMI (< 18.5 kg/m^2^, 18.5 to 24.9 kg/m^2^, 25.0 to 30.0 kg/m^2^, ≥ 30 kg/m^2^), education level, marital status, smoking status (never, former, now), history of malignancy, CVD, diabetes. To assess effect measure modification, we incorporated an interaction term into the model for each analysis. To examine linearity and investigate the shape of the dose–response relationship between LE8 and all-cause and CVD mortality in hypertensive patients, a Cox regression was conducted using a restricted cubic spline with 4 knots (5th, 35th, 65th, and 95th percentiles). The likelihood ratio test was employed to assess nonlinearity. To ensure the reliability of our findings, we conducted two sensitivity analyses. Firstly, in order to minimize the potential bias of reverse-causality, individuals who died within the initial 24 months of follow-up period were excluded. Secondly, we adopted the most recent guidelines from the AHA, which define hypertension as an SBP of ≥ 130 mmHg and/or DBP of ≥ 80 mmHg [13]. All the above statistical analyses were performed using R software (http://www.Rproject.org, version 4.1.2). Two-sided *P* < 0.05 was considered statistically significant.

## Results

### Baseline characteristics of study participants

**Fig. 2:**
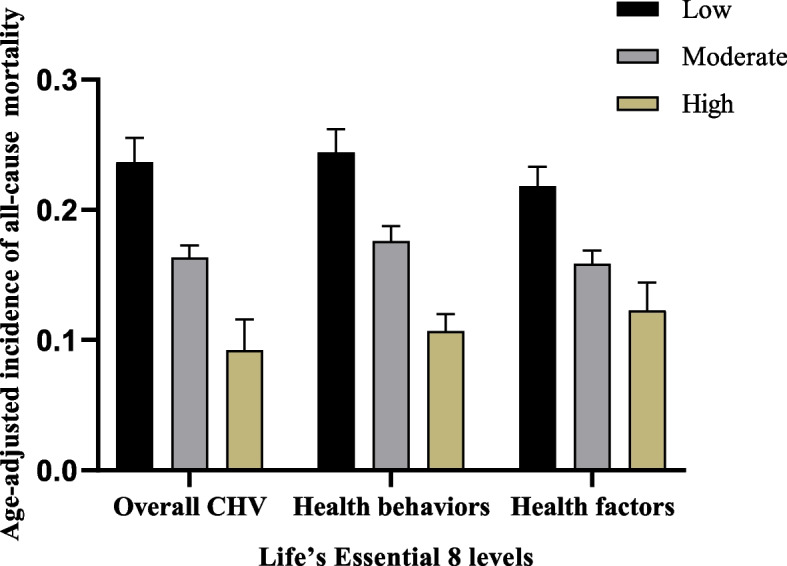
Incidence of age-adjusted all-cause mortality among hypertensive patients across various levels of Life’s Essential 8 scores

A total of 8,448 adults were included in the final analysis (weighted mean age, 57.58 years; 95% CI: 57.16–58.00 years), with 4,326 being female (weighted percentage, 51.21%; 95% CI: 49.91–52.50%). The weighted mean (standard error, SE) for LE8 was 60.13 ± 0.17. The demographic baseline characteristics of the participants included in the study were presented in Table 1, revealed marked differences in both baseline demographic and clinical features among participants categorized into three CVH groups. Furthermore, participants with high CVH exhibited a lower age-adjusted prevalence of all-cause mortality (9.23%, 95% CI: 6.87–11.60%) compared to those with moderate (16.34%, 95% CI: 15.41–17.26%) and low CVH (23.66%, 95% CI: 21.8–25.53%; Fig. 2). We discovered similar findings for health behaviors as well as health factors.

**Table 1:** Characteristics of adults participating in the National Health and Nutrition Examination Survey 2007–2016, categorized by Life's Essential 8 (LE8) score and weighted

Characteristic	Low (LE8 < 50)	Moderate (50 ≤ LE8 < 80)	High (LE8 ≥ 80)	*P*-value
Age, years	54.42 (53.73–55.10)	49.18 (48.65–49.72)	42.02 (41.12–42.93)	< 0.001
Age groups				0.9342
20–39	12.67 (10.87,14.72)	13.42 (12.24,14.70)	13.59 (10.05,18.14)	
40–59	39.22 (36.74,41.77)	39.01 (37.33,40.71)	40.90 (35.51,46.52)	
60–69	24.01 (21.35,26.89)	23.08 (21.56,24.67)	23.98 (19.84,28.68)	
70–79	14.74 (13.09,16.57)	15.37 (14.42,16.36)	13.63 (10.75,17.13)	
≥ 80	9.35 (7.87,11.08)	9.12 (8.22,10.11)	7.90 (5.93,10.45)	
Sex				< 0.001
Female	53.58 (50.87–56.27)	48.79 (47.87–49.71)	59.85 (57.85–61.83)	
Male	46.42 (43.73–49.13)	51.21 (50.29–52.13)	40.15 (38.17–42.15)	
Race				< 0.001
Non-Hispanic white	7.10 (5.24–9.54)	8.36 (6.79–10.24)	7.11 (5.86–8.62)	
Non-Hispanic black	15.84 (13.00–19.16)	10.83 (9.30–12.57)	5.91 (4.96–7.02)	
Mexican–American	67.74 (63.19–71.98)	69.45 (66.06–72.63)	72.81 (69.74–75.67)	
Others	9.32 (7.59–11.39)	11.37 (10.05–12.83)	14.17 (12.39–16.16)	
Education level				< 0.001
Less than high school	27.39 (24.69–30.26)	16.17 (14.73–17.72)	7.36 (6.32–8.55)	
High school or equivalent	30.21 (26.94–33.69)	24.40 (23.12–25.73)	12.16 (10.88–13.57)	
High school above	42.40 (39.25–45.62)	59.43 (57.19–61.64)	80.48 (78.36–82.44)	
Marital status				< 0.001
Unmarried	40.32 (37.59–43.11)	35.27 (33.76–36.81)	33.21 (30.92–35.59)	
Married	59.68 (56.89–62.41)	64.73 (63.19–66.24)	66.79 (64.41–69.08)	
Poverty income ratio				< 0.001
< 1.3	34.04 (30.89–37.34)	20.87 (19.17–22.69)	13.71 (11.89–15.75)	
1.3–1.5	40.32 (37.48–43.23)	37.05 (35.45–38.67)	30.31 (27.72–33.05)	
> 1.5	25.64 (22.08–29.55)	42.08 (39.62–44.58)	55.98 (52.52–59.38)	
BMI (kg/m^2^)				< 0.001
< 18.5	0.33 (0.15–0.72)	1.24 (1.04–1.48)	2.52 (1.97–3.22)	
18.5–24.9	6.46 (5.27–7.90)	20.97 (19.94–22.04)	58.07 (55.98–60.14)	
25–30	18.75 (16.80–20.88)	36.59 (35.46–37.75)	32.22 (30.40–34.11)	
≥ 30	74.45 (71.86–76.88)	41.20 (39.94–42.47)	7.18 (6.26–8.23)	
History of malignancy				< 0.001
No	88.01 (86.37–89.48)	88.47 (87.76–89.14)	92.33 (91.25–93.28)	
Yes	11.99 (10.52–13.63)	11.53 (10.86–12.24)	7.67 (6.72–8.75)	
Smoke status				< 0.001
0	24.52 (22.25–26.93)	51.62 (50.06–53.19)	79.76 (77.52–81.83)	
1	28.48 (26.39–30.66)	27.72 (26.47–29.01)	18.05 (16.24–20.00)	
2	47.00 (44.51–49.51)	20.66 (19.55–21.81)	2.19 (1.59–3.01)	
History of CVD^*^				< 0.001
No	78.77 (76.39–80.97)	91.16 (90.48–91.80)	97.45 (96.76–97.99)	
Yes	21.23 (19.03–23.61)	8.84 (8.20–9.52)	2.55 (2.01–3.24)	
History of diabetes				< 0.001
No	64.27 (61.69–66.78)	90.01 (89.37–90.61)	98.96 (98.46–99.29)	
Yes	35.73 (33.22–38.31)	9.99 (9.39–10.63)	1.04 (0.71–1.54)	
Total CVH score	42.14 (41.85–42.43)	66.18 (65.94–66.41)	86.78 (86.54–87.01)	< 0.001
HEI diet score	19.93 (18.73–21.13)	35.68 (34.79–36.57)	59.69 (58.28–61.10)	< 0.001
Physical activity score	26.10 (23.84–28.35)	72.36 (71.22–73.51)	95.44 (94.90–95.99)	< 0.001
Tobacco/nicotine exposure score	41.55 (39.38–43.71)	69.28 (68.10–70.47)	91.68 (90.60–92.76)	< 0.001
Sleep health score	66.00 (64.51–67.49)	82.78 (82.19–83.37)	92.69 (92.05–93.33)	< 0.001
Body mass index score	32.57 (30.91–34.23)	56.76 (55.92–57.61)	85.02 (84.12–85.92)	< 0.001
Blood lipids score	43.43 (41.80–45.07)	60.73 (59.97–61.48)	82.51 (81.53–83.50)	< 0.001
Blood glucose score	61.09 (59.68–62.51)	85.39 (84.87–85.90)	97.58 (97.15–98.01)	< 0.001
Blood pressure score	46.43 (44.93–47.92)	66.43 (65.57–67.29)	89.61 (88.80–90.42)	< 0.001
Physical activity, hour/week	0.50 (0.41–0.59)	2.40 (2.26–2.53)	4.86 (4.61–5.12)	< 0.001
Sleep health, hour/day	6.69 (6.58–6.72)	7.11 (7.09–7.21)	7.39 (7.28–7.44)	< 0.001
Blood lipids (non-HDL-C, mg/dL)	165.59 (163.13–168.08)	147.07 (145.73–148.49)	127.28 (125.66–128.92)	< 0.001
Fasting glucose, mg/Dl	124.91 (122.24–127.94)	109.48 (108.27–110.78)	98.08 (97.78–100.54)	< 0.001
HbA1c, %	6.19 (6.20–6.32)	5.58 (5.56–5.72)	5.28 (5.21–5.36)	< 0.001
All-cause mortality	15.67 (13.44,18.19)	10.55 (9.49,11.72)	5.52 (3.94,7.69)	< 0.001
CVD mortality	7.43 (5.96,9.22)	4.72 (4.08,5.46)	3.14 (1.86,5.25)	< 0.001

For continuous variables: survey-weighted mean (95% CI), P-value was by survey-weighted linear regression

For categorical variables: survey-weighted percentage (95% CI), P-value was by survey-weighted Chi-square test

^*^CVD included coronary heart disease, angina, myocardial infarction/heart attack, stroke, and heart failure

### Association between the LE8 and all-cause and CVD mortality

**Fig. 3:**
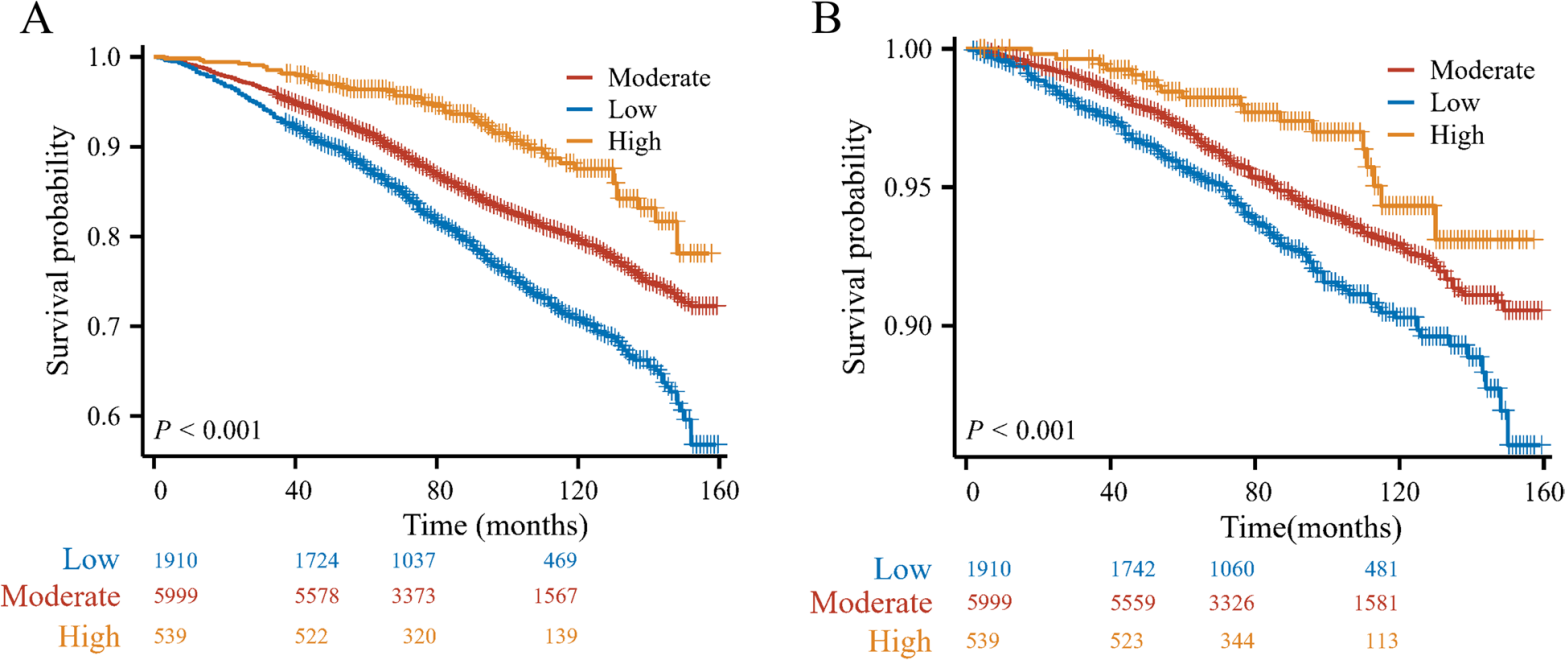
Kaplan–Meier plots of all-cause mortality **A** and cardiovascular disease-specific mortality **B** categorized by the total cardiovascular health metrics scores

During an average follow-up period of 7.41 years, a total of 1,482 (17.54%) participants died, with 472 deaths attributed to CVD. Overall, the low CVH group exhibited the highest risk for all-cause mortality (*P* < 0.001) (Fig. 3A) and CVD mortality (*P* < 0.001) (Fig. 3B). As presented in Table 2, when compared to the low CVH group, the high CVH group exhibited a reduced risk of all-cause mortality in the non-adjusted model (HR = 0.36, 95% CI: 0.27–0.48). This association remained stable after adjusting for socio-demographics and lifestyle factors in model I (HR = 0.61, 95% CI: 0.45–0.83), and further adjusting for health conditions in model II (HR = 0.63, 95% CI: 0.45–0.88). Moreover, negative associations were found between a 10-point increase in LE8 scores and mortality from all causes across all multivariable Cox regression models (Model I, HR = 0.91, 95% CI: 0.87–0.95; Model II, HR = 0.91, 95% CI: 0.86–0.96). After full multivariable adjustment, both moderate and high health behavior groups showed a reduced risk of all-cause mortality (all *P* < 0.05). A 10-point increase in the LE8 score corresponded to a HR of 0.92 (95%CI: 0.89–0.95) in relation to all-cause mortality.

**Table 2:** Association between Life’s Essential 8 score and all-cause and cardiovascular mortality of patients with hypertension in NHANES 2007–2016

	Model I	Model II	Model III
***All-cause mortality***			
Life’s Essential 8 score			
Low (0–49)	1(Reference)	1(Reference)	1(Reference)
Moderate (50–79)	0.67 (0.60, 0.74) < 0.0001	0.87 (0.77, 0.98) 0.0271	0.89 (0.78, 1.03) 0.1091
High (80–100)	0.36 (0.27, 0.48) < 0.0001	0.61 (0.45, 0.83) 0.0018	0.63 (0.45, 0.88) 0.0061
Per 10 points increase	0.82 (0.79, 0.85) < 0.0001	0.91 (0.87, 0.95) < 0.0001	0.91 (0.86, 0.96) 0.0004
Health behaviors score			
Low (0–49)	1(Reference)	1(Reference)	1(Reference)
Moderate (50–79)	0.69 (0.61, 0.77) < 0.0001	0.84 (0.75, 0.95) 0.0056	0.85 (0.74, 0.97) 0.0157
High (80–100)	0.39 (0.34, 0.46) < 0.0001	0.67 (0.57, 0.79) < 0.0001	0.67 (0.56, 0.81) < 0.0001
Per 10 points increase	0.85 (0.83, 0.87) < 0.0001	0.93 (0.91, 0.96) < 0.0001	0.92 (0.89, 0.95) < 0.0001
Health factors score			
Low (0–49)	1(Reference)	1(Reference)	1(Reference)
Moderate (50–79)	0.70 (0.63, 0.78) < 0.0001	0.88 (0.79, 0.99) 0.0271	0.89 (0.79, 1.01) 0.0604
High (80–100)	0.55 (0.44, 0.67) < 0.0001	0.78 (0.63, 0.98) 0.0297	0.80 (0.64, 1.01) 0.0597
Per 10 points increase	0.88 (0.85, 0.91) < 0.0001	0.94 (0.91, 0.97) 0.0003	0.95 (0.91, 0.98) 0.0025
***Cardiovascular mortality***			
Life’s Essential 8 score			
Low (0–49)	1(Reference)	1(Reference)	1(Reference)
Moderate (50–79)	0.72 (0.59, 0.87) 0.0010	0.72 (0.58, 0.90) 0.0032	0.76 (0.60, 0.97) 0.0298
High (80–100)	0.43 (0.26, 0.70) 0.0008	0.49 (0.30, 0.83) 0.0072	0.54 (0.31, 0.94) 0.0304
Per 10 points increase	0.84 (0.79, 0.90) < 0.0001	0.83 (0.77, 0.89) < 0.0001	0.82 (0.75, 0.90) < 0.0001
Health behaviors score			
Low (0–49)	1(Reference)	1(Reference)	1(Reference)
Moderate (50–79)	0.72 (0.59, 0.88) 0.0016	0.67 (0.54, 0.83) 0.0003	0.69 (0.54, 0.87) 0.0022
High (80–100)	0.53 (0.41, 0.68) < 0.0001	0.49 (0.37, 0.64) < 0.0001	0.50 (0.36, 0.69) < 0.0001
Per 10 points increase	0.89 (0.85, 0.93) < 0.0001	0.87 (0.83, 0.92) < 0.0001	0.87 (0.82, 0.92) < 0.0001
Health factors score			
Low (0–49)	1(Reference)	1(Reference)	1(Reference)
Moderate (50–79)	0.82 (0.68, 1.00) 0.0463	0.81 (0.66, 0.99) 0.0379	0.88 (0.71, 1.08) 0.2239
High (80–100)	0.66 (0.45, 0.95) 0.0266	0.68 (0.46, 1.00) 0.0471	0.76 (0.51, 1.13) 0.1730
Per 10 points increase	0.92 (0.87, 0.97) 0.0037	0.92 (0.87, 0.97) 0.0032	0.94 (0.88, 1.00) 0.0596

Model I adjust for: None

**Fig. 4:**
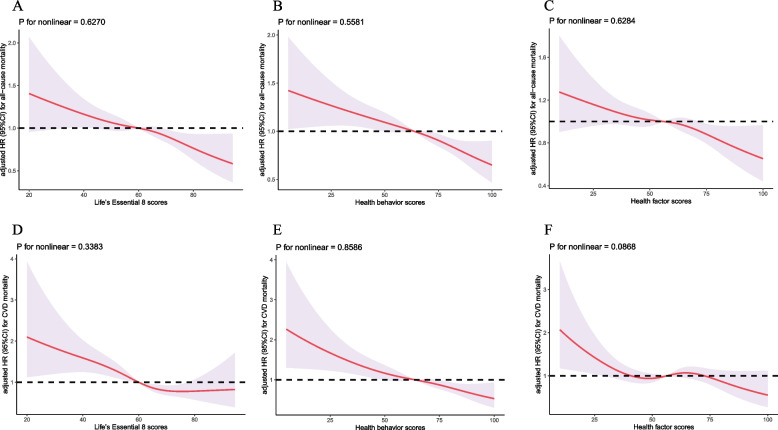
Dose–response relationships illustrating the association between ‘Life’s Essential 8’ scores, ‘Health Behavior’ score, ‘Health Factors’ Score, and their effects on all-cause **A**-**C **and cardiovascular disease-specific mortality **D**-**F**

Model II adjust for: sex, age, race/ethnicity, education level, marital status, PIR, BMI, waist circumference;

Model III adjust for: sex, age, race/ethnicity, education level, marital status, PIR, BMI, waist circumference, history of malignancy, history of CVD, history of diabetes, smoke status, DBP, and SBP;

In terms of CVD mortality, individuals with moderate or high CVH scores had a reduced risk of CVD mortality compared to those with low scores, with a 24% decrease (HR = 0.76, 95% CI: 0.60–0.97) and a 46% decrease (HR = 0.54, 95% CI: 0.31–0.94) respectively, after adjusting for all potential covariates. Additionally, for every 10-point increase in the LE8, the risk of CVD mortality decreased by 18% (HR = 0.82, 95% CI: 0.75–0.90). Similarly, individuals with moderate and higher scores of health behaviors had a reduced risk of CVD-specific mortality, with a 31% decrease (HR = 0.69, 95% CI: 0.54–0.87) and a 50% decrease (HR = 0.50, 95% CI: 0.36–0.69) respectively. Furthermore, for every 10-point increase in the health behaviors score, the risk of CVD mortality decreased by 13% (HR = 0.87, 95% CI: 0.82–0.92). Although the associations for health factors score did not reach statistical significance, similar trends were observed, with higher scores being linked to a reduced risk of CVD-specific mortality (*P* for trend < 0.05). Moreover, the restricted cubic spline analysis indicated a linear dose–response relationship between total CVH scores and both all-cause and CVD-specific mortality (*P* > 0.05 for non-linear association, as shown in Fig. 4). This means that as total CVH scores increase, the risk of both all-cause and CVD-specific mortality decreases in a linear manner.

Among 8448 hypertension patients, 6981 patients provided the onset age of hypertension. We use the patient's age minus the onset age of hypertension to calculate the duration of hypertension. We performed further analyses by hypertension duration (less than 5 years vs more than 5 years) to explore if the association between LE8 and mortality outcomes differs by the recency of hypertension diagnosis. The results showed that with the increase of CVH (from low to Moderate and then to high), the risk of all-cause death and cardiovascular death decreased (*P* for trend < 0.01). For patients with hypertension history of more than 5 years, the risk of all-cause death and cardiovascular death in high CVH group decreased more significantly (Table S2).

### Subgroup and sensitivity analysis

**Fig. 5:**
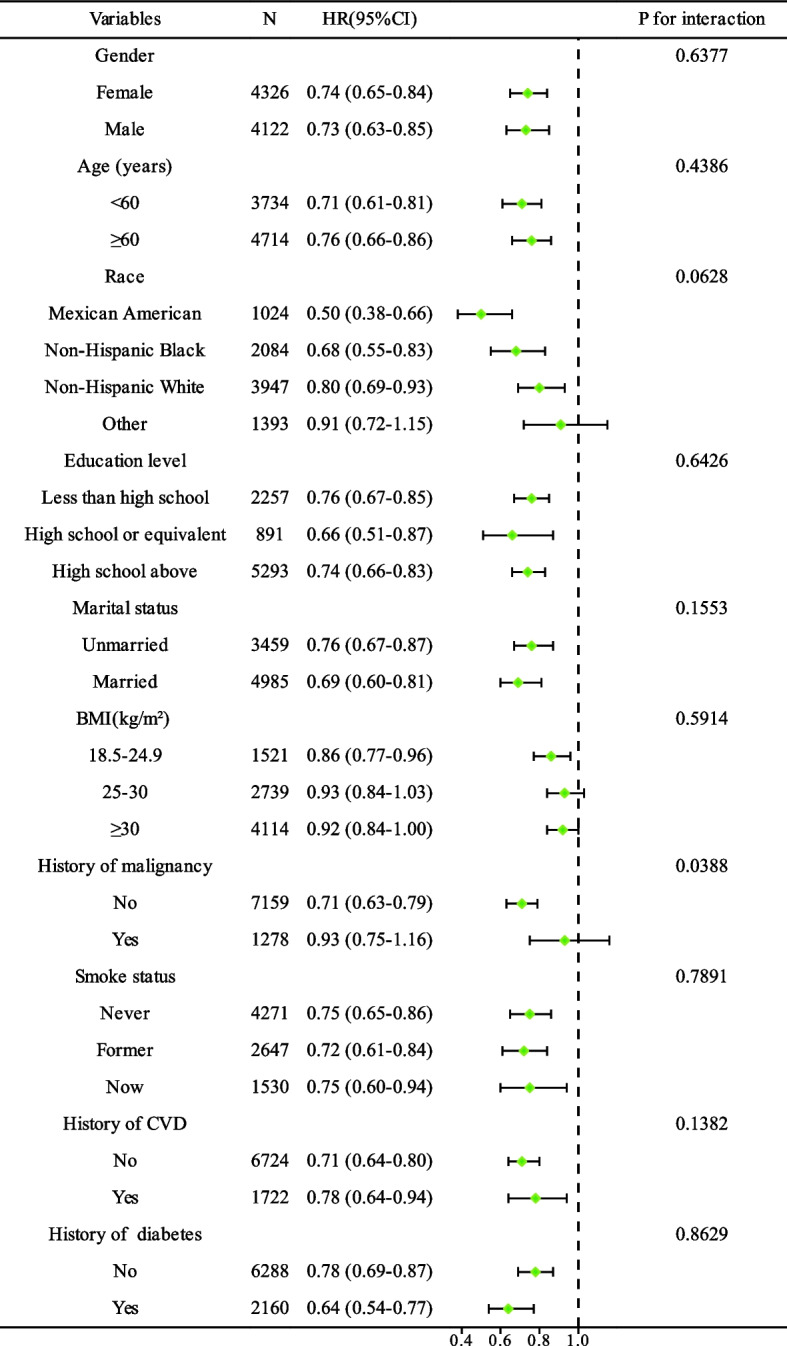
Stratified analyses of the impact of every 10-score increase in LE8 on all-cause mortality in hypertensive patients, considering potential modifiers

The relationship between LE8 and all-cause mortality, as revealed in the subgroup analysis, was consistent regardless of variations in age, sex, race, BMI, education level, marital status, smoking habits, history of cancer, history of CVD, and history of diabetes. However, despite the fact that the interactions for individuals with a history of cancer were less than 0.05, the similar directionality of these associations showed a downward trend in those without a history of cancer. This implies that such interactions might not have any substantial clinical relevance (Fig. 5).

The results remained consistent when several methods were utilized to verify the robustness of the results. First, we performed a sensitivity analysis by using a new hypertensive cutoff value of 130/80 mmHg according to the AHA guideline. The sensitivity analyses revealed that the primary outcome remained stable, with no significant changes observed after implementing the new diagnostic threshold for hypertension (Table S3). Furthermore, the relationship between LE8 and the risk of all-cause and CVD mortality in the fully adjusted model remained largely unchanged when excluding adult hypertensive participants who died within the initial 24 months of follow-up (Table S4).

## Discussion

The present study demonstrated the significant associations of LE8 with both all-cause and CVD-specific mortality in individuals with hypertension. Notably, individuals with a higher LE8 score, indicative of better CVH, showed a lower risk of all-cause and CVD mortality. The risk increase across the LE8 spectrum strongly highlights the importance of maintaining cardiovascular health throughout life.

A significant finding of our study was the protective effect of higher LE8 scores in individuals with hypertension. Hypertension has been widely recognized as a major risk factor for CVD [14], and it's been estimated that the population-attributable risk for death from coronary heart disease and stroke related to hypertension is approximately 45% and 51%, respectively [15]. Our study demonstrates that individuals with hypertension can reduce their risk of mortality by improving their LE8 scores. This suggests that comprehensive lifestyle modifications, as reflected by LE8, could be particularly beneficial in hypertensive individuals. These results match the AHA's strategic objectives, which pinpoint LE8 as a critical factor for preventing CVD [6].

The LE8 concept originated from western countries, but the findings support its relevance and importance in different contexts and populations, similar to the positive health effects seen in Japan through population-wide strategies [16]. Our results indicate that individuals with lower socioeconomic status had a higher risk of mortality, reflecting previous research emphasizing socioeconomic disparities in CVD outcomes [17]. Another significant aspect of our study is its focus on modifiable health behaviors and factors. Diet was a crucial component of LE8 in our study, a finding consistent with the Global Burden of Disease study identifying diet as a leading risk factor for deaths and disability-adjusted life-years globally [18]. Moreover, tobacco use and physical inactivity, two other components of LE8, are established risk factors for myocardial infarction worldwide [19]. The co-occurrence of these risk behaviors is common and highlights the importance of a comprehensive approach in promoting CVH [20].

The relevance of LE8 in various populations has been further substantiated by recent studies. A large prospective cohort study demonstrated that higher LE8 scores were significantly associated with lower risks of coronary heart disease, stroke, and overall cardiovascular disease. This study also highlighted that the LE8 model outperformed the previous Life's Simple 7 metrics, underscoring the enhanced predictive capability of the LE8, particularly with the inclusion of sleep health as a new component [21]. The findings from this study align with our results, reinforcing the importance of the LE8 in promoting cardiovascular health across diverse populations. Moreover, a study in China explored the age-specific associations of hypertension stages at diagnosis with cardiovascular and all-cause mortality among elderly patients. They found that advanced hypertension stages were significantly associated with higher risks of cardiovascular and all-cause mortality, particularly among older adults [22]. This study complements our findings by emphasizing the critical need for early and comprehensive cardiovascular health interventions.

Hypertension contributes to atherosclerosis development and progression, leading to various macrovascular complications [23]. High LE8 scores, indicative of good CVH, suggest well-controlled hypertension, among other health factors, which could, in turn, lower the risk for CVD and mortality. Components of health behaviors, such as regular physical activity, balanced diet, and non-smoking, are known to mediate the effects of hypertension on CVD [24]. They not only help in controlling blood pressure but also bring about improvements in other cardiovascular risk factors such as dyslipidemia, insulin resistance, and obesity [25]. Therefore, high LE8 scores reflecting healthier behaviors would naturally be linked with lower all-cause and CVD mortality. However, the precise mechanisms linking LE8 scores and mortality in hypertensive individuals are not fully understood and warrant further research. The interaction of genetic predisposition, environmental factors, and personal behaviors is complex and varies from individual to individual [26]. Understanding the precise biological pathways would require more targeted studies, possibly involving molecular and genetic analyses, to identify specific pathways that are influenced by the different components of LE8.

Our findings reinforce the importance of a healthy lifestyle in reducing mortality and improving population health. The association between LE8 and heart failure, another major cardiovascular event, has been previously shown, demonstrating its broader implications for cardiovascular health [27]. Interestingly, in our study, the relationships between LE8 and mortality risks remained robust irrespective of variations in sociodemographic and clinical factors. This observation supports the notion that LE8 can serve as a universal measure of CVH regardless of diverse backgrounds. We also noticed a similar trend of lower risk with higher LE8 scores across various subgroups. A cohort study also suggested that lifestyle changes could lead to significant improvements in atherosclerosis, further underlining the potential of modifying cardiovascular health factors [28]. Nevertheless, non-communicable diseases, including CVD, remain the leading cause of death worldwide [29]. Our study highlights the importance of LE8 as a potentially effective tool to monitor and improve CVH, with clear implications for disease prevention.

However, this study has limitations. First, it is important to note that this is an observational study, which could not prove causality. Future interventional studies investigating the effects of improving LE8 scores on the prognosis of hypertensive patients are needed to confirm our findings. Second, we were unable to account for all potential factors, which may confound the association between LE8 and mortality in hypertensive patients. However, our results are supported by the stability of subgroup analysis and sensitivity analysis. Besides, NHANES does not provide specific data regarding hypertensive emergencies or detailed hospital admission histories prior to the enrollment of participants. The absence of this data restricts our ability to directly assess the impact of prior hypertensive emergencies or admissions on the risk of all-cause and cardiovascular mortality in our cohort.

## Conclusions

Our research has demonstrated a negative correlation between higher LE8 scores, indicating healthier lifestyles and CVH, and the occurrence of both overall and CVD-specific mortality in individuals with hypertension. This suggests that initiatives aimed at promoting better CVH scores in public health and medical services could have significant implications in reducing mortality rates among hypertensive individuals.

### Supplementary Information

Supplementary Material 1.

Supplementary Material 2.

Supplementary Material 3.

Supplementary Material 4.

## Data Availability

Publicly available dataset was analyzed in this study. The National Health and Nutrition Examination Survey dataset are publicly available at https://www.cdc.gov/nchs/nhanes/index.htm.
