# Supplementary material for: Association between new Life’s Essential 8 and the risk of all-cause and cardiovascular mortality in patients with hypertension: a cohort study
Source: BMC Public Health. 2024 Jun 28;24:1730. doi: 10.1186/s12889-024-19189-z (PMC11212374; doi:10.1186/s12889-024-19189-z)
Supplement: Supplementary file 2 — Supplementary Material 2. [file 12889_2024_19189_MOESM2_ESM.docx]

**Table S2** Association between Life's Essential 8 Score and All-Cause and Cardiovascular Mortality in Hypertensive Patients Stratified by Hypertension Duration (NHANES 2007–2016)

|  | HP duration<5 years | HP duration>= 5 years | Total |
| --- | --- | --- | --- |
| All-cause mortality |  |  |  |
| Model I |  |  |  |
| Life’s Essential 8 score |  |  |  |
| Low (0–49) | 1 | 1 | 1 |
| Moderate (50–79) | 0.64 (0.52, 0.79) <0.0001 | 0.66 (0.56, 0.76) <0.0001 | 0.65 (0.58, 0.73) <0.0001 |
| High (80–100) | 0.29 (0.15, 0.55) 0.0001 | 0.35 (0.24, 0.52) <0.0001 | 0.33 (0.24, 0.47) <0.0001 |
| Per 10 points increase | 0.82 (0.76, 0.88) <0.0001 | 0.82 (0.78, 0.86) <0.0001 | 0.82 (0.79, 0.85) <0.0001 |
| Model II |  |  |  |
| Low (0–49) | 1 | 1 | 1 |
| Moderate (50–79) | 0.70 (0.55, 0.90) 0.0043 | 0.96 (0.81, 1.14) 0.6553 | 0.86 (0.75, 0.99) 0.0363 |
| High (80–100) | 0.61 (0.31, 1.20) 0.1526 | 0.54 (0.36, 0.82) 0.0038 | 0.55 (0.39, 0.78) 0.0009 |
| Per 10 points increase | 0.84 (0.77, 0.91) <0.0001 | 0.91 (0.86, 0.97) 0.0022 | 0.89 (0.85, 0.93) <0.0001 |
| LE8_10 |  |  |  |
| Model III |  |  |  |
| Low (0–49) | 1 | 1 | 1 |
| Moderate (50–79) | 0.83 (0.63, 1.09) 0.1719 | 0.97 (0.80, 1.17) 0.7212 | 0.91 (0.78, 1.06) 0.2196 |
| High (80–100) | 0.80 (0.39, 1.65) 0.5529 | 0.55 (0.35, 0.85) 0.0078 | 0.60 (0.42, 0.88) 0.0081 |
| Per 10 points increase | 0.89 (0.79, 1.00) 0.0411 | 0.90 (0.83, 0.96) 0.0026 | 0.89 (0.84, 0.94) <0.0001 |
| Cardiovascular mortality |  |  |  |
| Model I |  |  |  |
| Life’s Essential 8 score |  |  |  |
| Low (0–49) | 1 | 1 | 1 |
| Moderate (50–79) | 0.56 (0.38, 0.81) 0.0025 | 0.72 (0.55, 0.94) 0.0140 | 0.66 (0.53, 0.82) 0.0002 |
| High (80–100) | 0.48 (0.19, 1.21) 0.1204 | 0.41 (0.21, 0.80) 0.0086 | 0.43 (0.25, 0.73) 0.0020 |
| Per 10 points increase | 0.85 (0.74, 0.97) 0.0128 | 0.82 (0.75, 0.89) <0.0001 | 0.83 (0.77, 0.89) <0.0001 |
| Model II |  |  |  |
| Low (0–49) | 1 | 1 | 1 |
| Moderate (50–79) | 0.49 (0.32, 0.74) 0.0007 | 0.74 (0.55, 1.00) 0.0494 | 0.65 (0.52, 0.83) 0.0005 |
| High (80–100) | 0.45 (0.17, 1.20) 0.1119 | 0.50 (0.25, 0.99) 0.0467 | 0.48 (0.27, 0.84) 0.0100 |
| Per 10 points increase | 0.80 (0.68, 0.92) 0.0029 | 0.81 (0.73, 0.89) <0.0001 | 0.80 (0.74, 0.88) <0.0001 |
| Model III |  |  |  |
| Low (0–49) | 1 | 1 | 1 |
| Moderate (50–79) | 0.49 (0.31, 0.77) 0.0021 | 0.81 (0.58, 1.12) 0.2044 | 0.68 (0.52, 0.89) 0.0052 |
| High (80–100) | 0.44 (0.16, 1.27) 0.1289 | 0.58 (0.28, 1.21) 0.1472 | 0.52 (0.28, 0.95) 0.0322 |
| Per 10 points increase | 0.77 (0.64, 0.93) 0.0074 | 0.80 (0.71, 0.91) 0.0005 | 0.79 (0.71, 0.88) <0.0001 |

Model I adjust for: None

Model II adjust for: sex, age, race/ethnicity, education level, marital status, PIR, BMI, waist circumference;

Model III adjust for: sex, age, race/ethnicity, education level, marital status, PIR, BMI, waist circumference, history of malignancy, history of CVD, history of diabetes, smoke status, DBP, and SBP;
