# Supplementary material for: Association between new Life’s Essential 8 and the risk of all-cause and cardiovascular mortality in patients with hypertension: a cohort study
Source: BMC Public Health. 2024 Jun 28;24:1730. doi: 10.1186/s12889-024-19189-z (PMC11212374; doi:10.1186/s12889-024-19189-z)
Supplement: Supplementary file 3 — Supplementary Material 3. [file 12889_2024_19189_MOESM3_ESM.docx]

**Table S3** The association of LE8 with all-cause and cardiovascular mortality in individuals with hypertension defined by the novel diagnostic criteria (SBP/DBP: 130/80 mmHg)

|  | Non-adjusted | Adjust I | Adjust II |
| --- | --- | --- | --- |
| *All-cause mortality* |  |  |  |
| Life’s Essential 8 score |  |  |  |
| Low (0–49) | 1(Reference) | 1(Reference) | 1(Reference) |
| Moderate (50–79) | 0.61 (0.55, 0.68) <0.0001 | 0.58 (0.52, 0.66) <0.0001 | 0.72 (0.62, 0.82) <0.0001 |
| High (80–100) | 0.38 (0.29, 0.48) <0.0001 | 0.38 (0.29, 0.50) <0.0001 | 0.54 (0.40, 0.73) <0.0001 |
| Per 10 points increase | 0.80 (0.77, 0.83) <0.0001 | 0.77 (0.73, 0.80) <0.0001 | 0.83 (0.78, 0.88) <0.0001 |
| Health behaviors score |  |  |  |
| Low (0–49) | 1(Reference) | 1(Reference) | 1(Reference) |
| Moderate (50–79) | 0.66 (0.59, 0.73) <0.0001 | 0.64 (0.57, 0.72) <0.0001 | 0.72 (0.63, 0.82) <0.0001 |
| High (80–100) | 0.39 (0.34, 0.45) <0.0001 | 0.39 (0.33, 0.46) <0.0001 | 0.47 (0.39, 0.56) <0.0001 |
| Per 10 points increase | 0.85 (0.83, 0.87) <0.0001 | 0.83 (0.81, 0.86) <0.0001 | 0.85 (0.83, 0.88) <0.0001 |
| Health factors score |  |  |  |
| Low (0–49) | 1(Reference) | 1(Reference) | 1(Reference) |
| Moderate (50–79) | 0.86 (0.77, 0.95) 0.0047 | 0.85 (0.74, 0.96) 0.0116 | 1.00 (0.87, 1.16) 0.9651 |
| High (80–100) | 0.87 (0.73, 1.03) 0.1111 | 1.20 (0.97, 1.48) 0.1018 | 1.56 (1.22, 1.98) 0.0003 |
| Per 10 points increase | 0.96 (0.93, 0.99) 0.0046 | 0.98 (0.94, 1.02) 0.3430 | 1.06 (1.01, 1.12) 0.0308 |
| *Cardiovascular mortality* |  |  |  |
| Life’s Essential 8 score |  |  |  |
| Low (0–49) | 1(Reference) | 1(Reference) | 1(Reference) |
| Moderate (50–79) | 0.60 (0.50, 0.73) <0.0001 | 0.58 (0.47, 0.73) <0.0001 | 0.70 (0.55, 0.89) 0.0032 |
| High (80–100) | 0.34 (0.21, 0.54) <0.0001 | 0.40 (0.24, 0.66) 0.0004 | 0.52 (0.31, 0.90) 0.0182 |
| Per 10 points increase | 0.79 (0.74, 0.84) <0.0001 | 0.77 (0.71, 0.84) <0.0001 | 0.82 (0.74, 0.90) <0.0001 |
| Health behaviors score |  |  |  |
| Low (0–49) | 1(Reference) | 1(Reference) | 1(Reference) |
| Moderate (50–79) | 0.77 (0.63, 0.94) 0.0115 | 0.70 (0.56, 0.87) 0.0013 | 0.78 (0.61, 0.99) 0.0429 |
| High (80–100) | 0.49 (0.38, 0.64) <0.0001 | 0.48 (0.36, 0.64) <0.0001 | 0.55 (0.40, 0.76) 0.0003 |
| Per 10 points increase | 0.88 (0.84, 0.91) <0.0001 | 0.86 (0.82, 0.91) <0.0001 | 0.89 (0.84, 0.94) <0.0001 |
| Health factors score |  |  |  |
| Low (0–49) | 1(Reference) | 1(Reference) | 1(Reference) |
| Moderate (50–79) | 0.76 (0.64, 0.92) 0.0044 | 0.81 (0.65, 1.01) 0.0641 | 0.88 (0.69, 1.12) 0.2865 |
| High (80–100) | 0.54 (0.38, 0.78) 0.0008 | 0.82 (0.54, 1.26) 0.3626 | 0.95 (0.60, 1.50) 0.8252 |
| Per 10 points increase | 0.88 (0.84, 0.93) <0.0001 | 0.91 (0.84, 0.98) 0.0105 | 0.94 (0.86, 1.02) 0.1238 |

Non-adjusted model adjust for: None

Adjust I model adjust for: sex, age, race/ethnicity, education level, marital status, PIR, BMI, waist circumference;

Adjust II model adjust for: sex, age, race/ethnicity, education level, marital status, PIR, BMI, waist circumference, history of malignancy, history of CVD, history of diabetes, smoke status, DBP, and SBP;
