# Supplementary material for: Association between new Life’s Essential 8 and the risk of all-cause and cardiovascular mortality in patients with hypertension: a cohort study
Source: BMC Public Health. 2024 Jun 28;24:1730. doi: 10.1186/s12889-024-19189-z (PMC11212374; doi:10.1186/s12889-024-19189-z)
Supplement: Supplementary file 4 — Supplementary Material 4. [file 12889_2024_19189_MOESM4_ESM.docx]

**Table S4** Sensitivity analyses exploring the relationship between LE8 and the risk of all-cause and CVD mortality excluded participants died within two years of follow-up

|  | Model I | Model II | Model III |
| --- | --- | --- | --- |
| All-cause mortality |  |  |  |
| Life’s Essential 8 score |  |  |  |
| Low (0–49) | 1(Reference) | 1(Reference) | 1(Reference) |
| Moderate(50–79) | 0.66 (0.59, 0.75) <0.0001 | 0.90 (0.79, 1.03) 0.1136 | 0.93 (0.80, 1.07) 0.3065 |
| High (80–100) | 0.38 (0.28, 0.52) <0.0001 | 0.67 (0.48, 0.92) 0.0127 | 0.69 (0.49, 0.97) 0.0350 |
| Per 10 points increase | 0.82 (0.79, 0.86) <0.0001 | 0.92 (0.88, 0.97) 0.0009 | 0.92 (0.87, 0.98) 0.0069 |
| Health behaviors score |  |  |  |
| Low (0–49) | 1(Reference) | 1(Reference) | 1(Reference) |
| Moderate (50–79) | 0.73 (0.64, 0.82) <0.0001 | 0.90 (0.79, 1.02) 0.0943 | 0.91 (0.78, 1.05) 0.1825 |
| High (80–100) | 0.41 (0.35, 0.49) <0.0001 | 0.70 (0.59, 0.84) <0.0001 | 0.71 (0.58, 0.86) 0.0006 |
| Per 10 points increase | 0.86 (0.84, 0.88) <0.0001 | 0.94 (0.92, 0.97) <0.0001 | 0.93 (0.90, 0.97) 0.0001 |
| Health factors score |  |  |  |
| Low (0–49) | 1(Reference) | 1(Reference) | 1(Reference) |
| Moderate (50–79) | 0.71 (0.63, 0.79) <0.0001 | 0.89 (0.79, 1.00) 0.0523 | 0.89 (0.79, 1.02) 0.0860 |
| High (80–100) | 0.54 (0.43, 0.68) <0.0001 | 0.79 (0.62, 0.99) 0.0439 | 0.80 (0.63, 1.01) 0.0650 |
| Per 10 points increase | 0.88 (0.85, 0.91) <0.0001 | 0.95 (0.91, 0.98) 0.0014 | 0.95 (0.91, 0.98) 0.0050 |
| Cardiovascular mortality |  |  |  |
| Life’s Essential 8 score |  |  |  |
| Low (0–49) | 1(Reference) | 1(Reference) | 1(Reference) |
| Moderate (50–79) | 0.73 (0.60, 0.89) 0.0022 | 0.73 (0.59, 0.91) 0.0056 | 0.77 (0.60, 0.99) 0.0429 |
| High (80–100) | 0.43 (0.27, 0.71) 0.0009 | 0.51 (0.30, 0.85) 0.0096 | 0.56 (0.32, 0.97) 0.0377 |
| Per 10 points increase | 0.84 (0.79, 0.90) <0.0001 | 0.83 (0.77, 0.90) <0.0001 | 0.83 (0.75, 0.91) <0.0001 |
| Health behaviors score |  |  |  |
| Low (0–49) | 1(Reference) | 1(Reference) | 1(Reference) |
| Moderate (50–79) | 0.72 (0.59, 0.89) 0.0018 | 0.67 (0.54, 0.84) 0.0003 | 0.69 (0.54, 0.87) 0.0021 |
| High (80–100) | 0.53 (0.41, 0.69) <0.0001 | 0.49 (0.37, 0.65) <0.0001 | 0.50 (0.36, 0.69) <0.0001 |
| Per 10 points increase | 0.89 (0.85, 0.93) <0.0001 | 0.87 (0.83, 0.92) <0.0001 | 0.87 (0.82, 0.92) <0.0001 |
| Health factors score |  |  |  |
| Low (0–49) | 1(Reference) | 1(Reference) | 1(Reference) |
| Moderate (50–79) | 0.84 (0.69, 1.01) 0.0690 | 0.83 (0.68, 1.01) 0.0645 | 0.90 (0.72, 1.11) 0.3088 |
| High (80–100) | 0.67 (0.46, 0.97) 0.0345 | 0.70 (0.47, 1.02) 0.0651 | 0.78 (0.52, 1.16) 0.2150 |
| Per 10 points increase | 0.93 (0.88, 0.98) 0.0056 | 0.92 (0.87, 0.98) 0.0059 | 0.95 (0.89, 1.01) 0.0816 |

Non-adjusted model adjust for: None

Adjust I model adjust for: sex, age, race, education level, marital status, PIR, BMI, waist circumference;

Adjust II model adjust for: sex, age, race, education level, marital status, PIR, BMI, waist circumference, history of malignancy, history of CVD, history of diabetes, smoke status, DBP, and SBP;
